# Parental Involvement in Children’s Sleep Care and Nocturnal Awakenings in Infants and Toddlers

**DOI:** 10.3390/ijerph17165808

**Published:** 2020-08-11

**Authors:** Benedetta Ragni, Simona De Stasio

**Affiliations:** Department of Human Studies, LUMSA University, 00193 Rome, Italy; b.ragni@lumsa.it

**Keywords:** sleep, paternal involvement, toddlers, infants

## Abstract

Background: Sleep regulation and consolidation represent critical developmental processes that occur in the first years of life. Recent studies have highlighted the contribution of caregivers to sleep development. However, the majority of them have primarily focused on maternal behaviors, overlooking fathers. The main goal of the present study was to investigate the associations between paternal and maternal involvement in children’s sleep care and the number of night awakenings reported by both parents in infants and toddlers. Methods: One-hundred-and-one families of infants aged 8 to 12 months and 54 families of toddlers aged 18 to 36 months filled out the following self-report questionnaires: The Brief Infant Sleep Questionnaire and an ad hoc questionnaire to assess parental involvement in sleep care for children. A moderate actor–partner interdependence (APIM) with path analysis was performed to test the predictive role of parental involvement on the children’s sleep (no. of nocturnal awakenings) and the moderation role of age on these relationships. Results: Paternal involvement in children’s sleep care was associated with the number of night awakenings reported by both parents. Moreover, a significant interaction effect emerged between the children’s age and paternal involvement in children’s sleep care for predicting nocturnal awakenings. Conclusions: The main outcomes of this study point to the protective role of paternal involvement in children’s sleep during the first years of life.

## 1. Introduction

Sleep regulation and consolidation represent critical and complex developmental processes that occur in the first years of life [1]. Although most infants are able to fall asleep independently and back to sleep following night wakings by 12 months of age [2,3], 25–50% of 6–12-month-old infants and 50% of toddlers rely on caregiver intervention and experience problematic night wakings [4].

Findings from longitudinal and large cohort studies, guided by the transactional model [5,6], have highlighted the contribution of caregivers’ behaviors and parent–child interactions to sleep development [2]. However, the majority of these studies have primarily focused on maternal behaviors, overlooking fathers and their role in children’s sleep [7].

This lack of research on fathers is surprising, considering that most of the father–child interactions occur in the evening when fathers return from work and that the growing evidence suggests that paternal involvement in childcare has a positive influence on the development of children [8,9,10].

Research and clinical experience suggest that, during the first three years of life, fathers are less involved than mothers regarding putting their children to sleep and less likely to approach their infants during the night [11,12,13]. However, when fathers are reported to be more involved in their children’s care, infants and toddlers display more consolidated sleep patterns [7,14,15,16]. With regard to infants, Tikotzky et al. [7,13] found that paternal involvement in infants’ general and sleep care at 1, 3, and 6 months of life was related to a lower number of night awakenings. Moreover, Ragni et al. [17] found that paternal bedtime involvement at 8–12 months was related to lower bedtime difficulties perceived by both parents. Concerning toddlers, the 1–3-year-olds of fathers that were less involved in general [15] and with bedtime child caregiving specifically [16] had more sleep problems, and sleep duration at three years of life was predicted by paternal emotional support and evocation of the child [14].

Hence, it may be that dividing caregiving tasks between parents helps mothers to achieve better sleep, which in turn, may contribute to greater consolidation of infant sleep [7]. Furthermore, recent studies demonstrated that fathers interact differently with their infants at night, and this may promote the consolidation and regulation of infant sleep [18,19,20]. Fathers have different perceptions regarding infant sleep than mothers [18,21]. They are more likely to endorse a limit-setting approach (they are more likely to set limits and resist the infant’s demands, emphasizing that the child should learn self-soothing with minimal parental help), and they find it easier to reduce active soothing (e.g., feeding, cuddling) at night [19,20,22].

This corpus of research represents a significant advance in our knowledge about the important role of fathers in infant sleep. However, these findings are partial and more studies are needed in order to corroborate them. 

First, no previous studies have examined the association between fathers’ involvement in children’s sleep care and sleep regulation at 8–12 months of age. According to the *Diagnostic Classification of Mental Health and Developmental Disorders of Infancy and Early Childhood (DC: 0–5)* [23], from the age of 8 months onward, children could develop night-waking disorders. Indeed, although at 6–9 months, infants should start to transition between wakefulness and sleep independently, at 12 months, there is a subset of them who continue to signal upon waking and require parental assistance to fall back to sleep [2]. Thus, examining the relationships between fathers’ involvement in children’s sleep care and night awakenings reported by parents at 8–12 months could advance our understanding of paternal involvement in the onset and maintenance of sleep problems, and could inform intervention programs.

Second, no studies have investigated potential differences in paternal sleep involvement between infants and toddlers. Following childbirth, parents often encounter significant changes in their physical and psychosocial health. Several studies have highlighted the role of partners in buffering children’s development from the impact of parental psychosocial functioning, especially in terms of mental health and affective disorders, which arise during the first 12 months postpartum [24]. Indeed, fathers represent an important source of support and assistance for mothers during the first months postpartum [25]. For these reasons, it may be hypothesized that the protective role of paternal involvement in sleep care and children’s sleep quality could be stronger during the first year of life than during toddlerhood.

Finally, previous studies had not included both maternal and paternal involvement in children’s sleep care into a unique predictive model of children’s sleep. If we take children’s sleep to be a familial phenomenon, it is important to include both maternal and paternal involvement to take into account the nonindependence between fathers and mothers as partners in the couple [26]. Indeed, the partners in a couple can affect each other’s behaviors reciprocally and differentially, and may be affected similarly or differently by distal factors [26,27]. Concerning sleep, it could be possible that involved fathers influence maternal behaviors and encourage them to reduce nighttime practices associated with less consolidated sleep [28,29]. Moreover, it could also be possible that supported mothers, independently from the fathers’ influence, behave differently with their infants, encouraging their autonomy, than mothers who assume sole responsibility for infant care [28].

### The Current Study

In light of these limitations and challenges, the main goal of the present study was to investigate the associations between paternal and maternal involvement in children’s sleep care and the number of night awakenings reported by both parents in infants (8–12 months) and toddlers (18–36 months).

Specifically, we hypothesized that greater paternal involvement in children’s sleep care would predict a decrease in the number of night awakenings reported by both parents (H1). Second, according to the literature, we expected that greater maternal involvement in children’s sleep care would predict an increase in the number of night awakenings reported by both parents (H2). Finally, we hypothesized that children’s age would moderate the association between parental involvement and children’s sleep, and specifically, that this association was stronger for infants than for toddlers (H3).

To the best of our knowledge, this is the first study that investigated relationships among the studied variables in an Italian sample. The involvement of fathers in child caregiving and the divisions of family roles are influenced by cultural ideologies, expectations, and norms [30,31]. Italy could be an interesting context for this investigation due to its gender system, which is characterized by a low level of involvement of fathers in childcare [32].

The last few decades have shown a progressive reduction in the gender imbalance in childcare; however, these changes have not affected all types of care, and mothers continue to carry the main responsibilities [33].

Italian fathers are more likely to think that mothers are responsible for coping with tasks related to childcare, especially with affective tasks [8]. In a recent study of Tanturri and colleagues [34], Italian fathers have been compared to fathers in Sweden, France, and the UK. Results from this study showed that in Italy and France, fathers show the lowest commitment to fathering activities. They spend more time in non-childcare activities (that do not always require strong commitment), and they tend to share this time with partners, thus further lowering their “burden” of child-rearing [34].

In addition to this, Italian public policies are less strongly oriented to gender equality. With regard to paternity leave, employed Italian fathers are only entitled to one day of paternity leave, and only since 2012. Moreover, fathers can take two additional days, but mothers need to agree to transfer these days from their maternity leave allocation, and these leave days should be used within five months after the child’s birth. It is evident that the introduction of paternity leave is still symbolic, and that Italian fathers are more reluctant to take it up because they do not find a social environment that supports them in this choice [34].

Therefore, this study also offers an opportunity to examine the presence of differences in parental involvement in children’s sleep care in a new cultural context, as characterized by a persistent gender gap [9]. 

## 2. Materials and Methods

### 2.1. Participants

One-hundred-and-fifty-five Italian heterosexual couples were recruited. One-hundred-and-one were families of infants aged 8 to 12 months (M = 10.67, SD = 2.12) and 54 were families of toddlers aged 18 to 36 months (M = 29.44, SD = 5.42). The criteria for participation were full-term pregnancy, absence of hospitalizations lasting more than a week, and the absence of any physical/mental disability. The mean age for parents in our sample was 36 years (SD = 4.7) for males and 36 years (SD = 4.3) for females. Considering the levels of education, 48% of mothers and 32% of fathers had a degree or higher, 40% of mothers and 43% of fathers had completed high school, and 12% of mothers and 25% of fathers had completed middle school or less. Parents were recruited from kindergartens located in Rome, Italy.

They received the questionnaires from trained psychologists who invited parents to complete them independently at home. The couples were provided with a prepaid envelope, which they used to return the questionnaires. Participants received written information on Italian privacy regulations and signed an informed consent form. Measures were completed at home. Data were gathered from September 2016 to June 2019. The study was approved on 1 September 2016 by the Ethics Committee for Scientific Research of “Libera Università Maria Ss. Assunta” (LUMSA University) of Rome, Italy.

### 2.2. Instruments

#### 2.2.1. Parental Involvement

Parental perceptions of their personal and their partner’s involvement in sleep caring for children were evaluated using an ad hoc questionnaire created by the authors comprising two single items, rated on a five-point Likert scale (e.g., “How much are you involved in your children’s sleep care?” and “How much is your partner involved in your child’s sleep care?”). Higher scores indicated higher perceived involvement of parents in children’s sleep care in terms of taking responsibility for it.

Specifically, for this study, we used parental involvement in children’s sleep care as perceived by their partner as independent variables, where such cross-informant data may increase the reliability of information about individual functioning within the dyad [35].

#### 2.2.2. The Expanded Version of the Brief Infant Sleep Questionnaire (BISQ [36])

The BISQ is a self-report measure that includes specific questions about infant nighttime sleep patterns, sleep-related behaviors, sleeping arrangements, bedtime routines, and other parental interventions. As in other studies [37,38,39], we selected the following BISQ item as an indicator of sleep regulation: “How often does your child wake during the night, if ever?” and we asked fathers and mothers to answer it. Fewer night wakings may be used as crude indicators of self-regulated sleep, as children who soothe themselves to sleep spend less time awake at night [1]. The BISQ has been validated against actigraphy and daily logs with significant correlations between sleep onset (*p* < 0.001 for both), sleep duration (*p* < 0.05 for both), and night wakings (*p* < 0.0001) [40]. Moreover, its sensitivity in documenting infant sleep and the effects of environmental factors has been well established [36].

### 2.3. Data Analyses

Means and standard deviations were reported for the variables under study, and Student’s *t*-test was performed in order to investigate differences between the two groups. Pearson bivariate correlations were calculated for mothers and fathers. 

**Figure 1:**
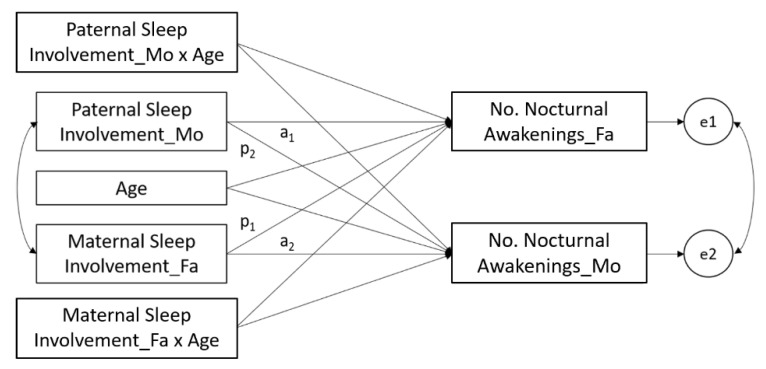
Moderated actor–partner interdependence model (APIM) model. Mo: Reported by mothers; Fa: Reported by fathers; No.: Number; a_1_ and a_2_: Actor effects; p_1_ and p_2_: Partner effects; e1 and e2: Measurement errors

By considering children’s sleep as a familial phenomenon and in order to adequately explain sources of variability among the studied variables, a moderated actor–partner interdependence model (APIM) [41,42] with path analysis was performed to test the predictive role of parental involvement on children’s sleep and the moderation role of age on these relationships. Consistent with recommendations by Kenny et al. [41], we modeled data at the couple level, with the number of night awakenings reported by mothers and fathers as covarying outcomes. We tested a model, shown in Figure 1, that reflected the number of night awakenings reported by mothers and fathers as the final dependent variables, paternal involvement in children’s sleep as perceived by mothers, and maternal involvement in children’s sleep as perceived by fathers as the independent variables; the children’s age was the between-dyad moderator. Analyses were performed with MPlus 8.0 [43].

## 3. Results

Descriptive statistics and bivariate correlations between the studied variables are reported in Table 1. Mothers were more involved in children’s sleep care than fathers, both for the infants’ (*t*(308) = −9.717, *p* = 0.000) and toddlers’ groups (*t*(106) = −3.805, *p* = 0.000). Moreover, mothers of infants were significantly more involved in their children’s sleep care than the mothers of toddlers (Table 1). In addition, the number of night awakenings reported by both mothers and fathers was greater for infants than for toddlers (Table 1).

Results from the bivariate correlations demonstrated suitably moderate associations between the variables included in the final model (Table 1). A negative association emerged between the maternal and paternal involvements in children’s sleep care (*r* = −0.24, *p* < 0.01).

### Moderated APIM Analyses

Table 2 presents a summary of the APIM analyses performed. For the main effects, a significant actor effect was found in the relationship between parental involvement in children’s sleep care and the number of nocturnal awakenings for fathers (a_1_). Specifically, we found a negative relationship between paternal involvement in children’s sleep care and the number of night awakenings reported by fathers (*b* = −0.338, *p* = 0.000). Moreover, a partner effect was found that indicated a negative association between paternal involvement in children’s sleep care and the number of night awakenings reported by mothers (p_2_) (*b* = −0.326, *p* = 0.000). 

**Figure 2:**
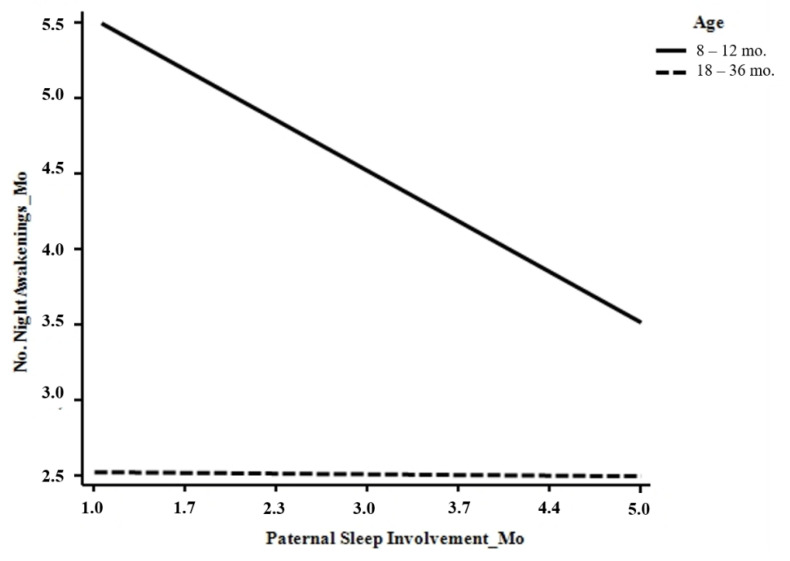
Links between paternal sleep involvement and the number of night awakenings reported by mothers.

**Figure 3:**
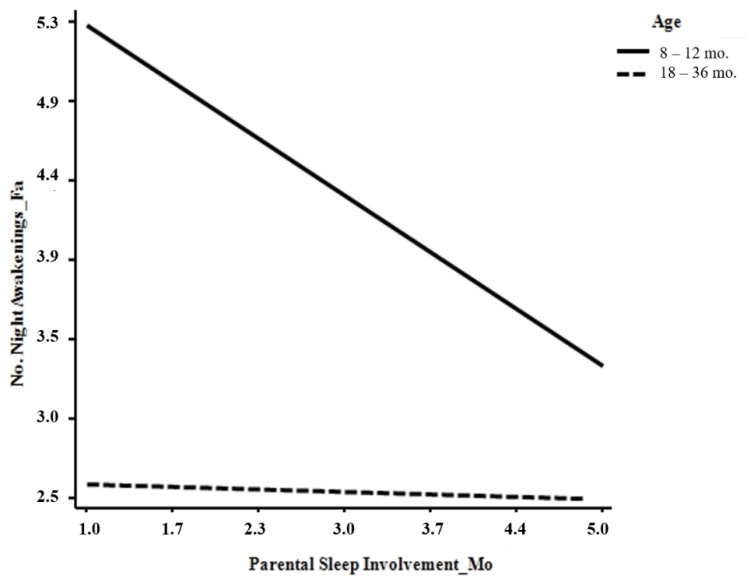
Links between paternal sleep involvement and the number of night awakenings reported by fathers.

Children’s age was negatively associated with the number of night awakenings reported both by mothers (*b* = −1.069, *p* = 0.005) and fathers (*b* = −1.167, *p* = 0.003). When considering the moderating effects of age, a significant interaction effect emerged between children’s age and paternal involvement in children’s sleep care in predicting the number of nocturnal awakenings reported both by mothers (*b* = 0.512, *p* = 0.012) and fathers (*b* = 0.536, *p* = 0.012). In particular, by decomposing these effects using a simple slope analyses, we found that the negative association between paternal involvement in children’s sleep care and the number of night awakenings reported by both parents increased only for mothers (*b* = −0.510, *t* = −3.979, *p* = 0.000) and fathers (*b* = −0.501, *t* = −3.741, *p* = 0.000) of infants aged 8–12 months and not for mothers (*b* = −0.007, *t* = −0.046, *p* = 0.962) and fathers (*b* = −0.021, *t* = −0.134, *p* = 0.893) of toddlers (Figure 2 and Figure 3).

## 4. Discussion

Findings from our study showed that Italian mothers were more involved in their children’s sleep care than fathers, in particular with younger children (8–12 months). Although the last few decades have shown a progressive reduction of the gender imbalance in childcare, in the Italian context, these changes have not affected all types of care, and mothers continue to carry the main responsibilities [34]. According to Tikotzky et al. [13], we may hypothesize that the differences in parental nighttime behaviors are determined by several factors. First, mothers reported a lower level of parental crying tolerance than fathers [44]; therefore, they may be more activated by the crying of their infant [45] and may respond faster to their infants during the night. In addition, a recent study by Reader and colleagues [21] had found that mothers endorsed stronger beliefs about responding to infant night awakenings than fathers. Instead, fathers, in comparison to mothers, endorse a limit-setting approach that enhances infants’ self-soothe ability [18]. Moreover, regarding nighttime practices, some mothers may believe that they are more competent at soothing the infant back to sleep than fathers [46].

With regard to the main results of our study, the findings confirmed our first hypothesis (H1). In agreement with previous studies [7,13,16], we found that when fathers are involved in their children’s sleep care, the number of night awakenings reported by both mothers and fathers decreased, even in infants aged 8–12 months. Several studies had suggested that sleep–wake regulation at night may also be related to paternal characteristics [15]; however, none of them had investigated these associations at this age. Although at 6–9 months, infants should start to transition between wakefulness and sleep independently, at 12 months, there is a subset of them who continue to signal upon waking and require parental assistance to fall back to sleep [2]. Our findings offer support to previous studies, suggesting a protective role of fathers’ involvement in children’s sleep care also for 8-to-12-months-old infants’ sleep regulation.

There are several possible explanations for these associations. On the one hand, it may be that paternal involvement influences child’s sleep through the fathers’ tendency to use a higher degree of a limit-setting approach that encourages children to self-regulate during the night [18]. Thus, higher paternal involvement can help fathers set limits around bedtime and thereby reduce night waking. On the other hand, fathers’ involvement may affect children’s development indirectly [47] through influencing mother–child interactions and supporting the mother’s decisions and behaviors.

With regard to maternal involvement in children’s sleep care, our findings did not confirm our second hypothesis (H2). In our sample, we did not find a significant association between maternal involvement in children’s sleep care and the number of night awakenings reported by both parents. These results are in contrast with previous literature showing greater maternal involvement in children’s sleep care as a risk factor for children’s self-soothe ability and their sleep regulation and consolidation [29]. We may hypothesize a difference between maternal involvement, in terms of bedtime and nighttime care as investigated in this study, and greater maternal involvement, in terms of bedtime and nighttime practices, which interfere with children’s self-regulation ability, in predicting children’s sleep. More studies are needed in order to deepen these results.

To the best of our knowledge, this is the first study that included both maternal and paternal involvement in children’s sleep care into a unique predictive model of children’s sleep. If we take children’s sleep to be a familial phenomenon, it is important to include both maternal and paternal involvement to take into account the nonindependence between fathers and mothers as partners in the couple [26]. Indeed, the partners in a couple can affect each other’s behaviors reciprocally and differentially, and may be affected similarly or differently by distal factors [26,27]. Research has suggested that paternal involvement may have a positive effect on parental well-being and family functioning. For example, paternal involvement in child-caregiving responsibilities was found to be a central buffer against maternal distress and depression during the transition to parenthood [25,48]. Indeed, high paternal involvement may relate to a better emotional climate of the family [49] and thus provide a better source of emotional and instrumental support for the mothers [50]. Concerning sleep, it could be possible that involved fathers influence maternal behaviors and encourage them to reduce nighttime practices associated with less consolidated sleep [28,29]. Moreover, it could be that couples who share infant caregiving are perhaps characterized by higher levels of support and lower levels of stress, which could exert a calming and positive influence on the sleep of the infant. It may also be possible that couples who share infant caregiving during the day hold similar expectations about nighttime behavior. These expectations, if transferred consistently to the infant, may facilitate the process of achieving consolidated sleep.

By considering children’s age and interaction effects, our third hypothesis (H3) was partially confirmed. Children’s age moderated the association between paternal involvement in children’s sleep and the number of night awakenings; however, a significant effect emerged only for infants aged 8–12 months. These findings are in contrast with our previous studies [16] in which we found that paternal involvement in bedtime caring predicted a decrease in the number of night awakenings in children aged 1–3 years old. However, in their longitudinal study with fathers of 3-years-old children, Bernier, and Carrier [14] found that among the six dimensions of paternal involvement pertaining to the relationship with the child investigated, the three that were related to child sleep at 3 years of age were the more closely related to emotional aspects of parent–child relationships: basic child care, emotional support, and evocation. Thus, we may speculate that during the postpartum period, dividing tasks related to children’s sleep could have a stronger effect on mothers as a buffer against distress and depression during the transition to parenthood. During toddlerhood, when paternal involvement increases, fathers’ emotional engagement with their children may be a key aspect in the development of young children’s sleep patterns. These results should be interpreted with caution. More studies are needed in order to deepen the role of different aspects of fathering in relation to child sleep, especially those dimensions of fathering that entail a strong emotional component [15].

This study presents several limitations. The modest sample size precluded more sophisticated analyses, and objective sleep measures would provide a more reliable assessment of children’s sleep quality. Moreover, although we used cross-informant data, paternal and maternal involvement was only measured with a two-item self-reported questionnaire. Furthermore, we did not investigate dimensions (e.g., basic child care or emotional support) and behaviors (e.g., reading a book before bed, or comforting the child when he/she wakes up during the night, etc.) related to parental involvement in children’s sleep, both at bedtime and during the night.

Further longitudinal research is needed to corroborate and clarify whether these associations are causal and the role of other demographic characteristics (e.g., parental education and occupation, parents’ age, family size, etc.) in influencing these associations. Furthermore, future research is needed to provide more in-depth investigate regarding which aspects of maternal and paternal involvement is more crucial for infants’ and toddlers’ sleep, through which pathways (e.g., its relationship with co-parenting quality), and whether paternal involvement in children’s sleep could be potentiated with evidence-based intervention.

## 5. Conclusions

The main outcomes of this study point to the protective role of paternal involvement in children’s sleep during the first years of life. Although Italian mothers were more dominant in children’s sleep caregiving, many fathers do take part in it. When mothers reported them to be more involved, the number of night awakenings perceived by both parents decreased.

These results have important implications. On the one hand, this study contributes to enhancing the understanding of risk and protective factors for children’s sleep quality among families, healthcare providers, and the general public. Indeed, it highlighted the role of fathers in children’s sleep care as a potential protective factor for night-waking disorders. This result could usually inform Italian public policies, which are less strongly oriented to gender equality.

On the other hand, with regard to the clinical practice, this study raises awareness of both the assessment and treatment of children’s sleep problems. According to the literature guided by the transactional model [5,6], clinicians should take the onset and maintenance of children’s sleep problems to be a familial phenomenon. They should investigate parental factors and caregiving behaviors as possible intervening factors in sleep problems. Specifically, according to our results, they should work with families in order to include fathers and increase their involvement in children’s sleep care.

## Figures and Tables

**Table 1:** Correlations, means, standard deviations, and *t*-test of the study variables.

Variables	1	2	3	4	5	Infants (*n* = 101) M (SD)	Toddlers (*n* = 54) M (SD)	*p* (*t*-test)
1. Children’s age	1					10.67 (2.12)	29.44 (5.42)	
2. Paternal sleep involvement_Mo	0.08	1				3.10 (1.05)	3.22 (1.20)	0.511
3. Maternal sleep involvement_Fa	−0.15	−0.24 **	1			4.34 (0.73)	4.04 (1.01)	0.036
4. No. of night awakenings_Mo	−0.58 **	−0.23 **	0.18 *	1		4.46 (1.59)	2.46 (0.98)	0.000
5. No. of night awakenings_Fa	−0.52 **	−0.23 **	0.07	0.88 **	1	4.26 (1.67)	2.55 (0.96)	0.000

** *p* < 0.01; * *p* < 0.05; M = Mean; SD = Standard deviation; *n* = Sample size.

**Table 2:** APIM results: Associations between paternal and maternal involvement in children’s sleep care with the number of night awakenings with the moderating context of age.

Parameters	Estimate (*b*)	*p*
**Main Effects**		
Actor Effects of Sleep Involvement		
PSI _Mo → NW_Fa (a_1_)	−0.338	0.000
MSI_Fa → NW_Mo (a_2_)	0.025	0.737
Partner Effects of Sleep Involvement		
PSI _Mo → NW_Mo (p_2_)	−0.326	0.000
MSI_Fa → NW_Fa (p_1_)	−0.044	0.570
Age → NW_Mo	−1.069	0.005
Age → NW_Fa	−1.167	0.003
**Interaction Effects**		
Actor Sleep Involvement by Age		
PSI_Mo × Age → NW_Fa	0.536	0.012
MSI_Fa × Age → NW_Mo	0.065	0.819
Partner Involvement by Age		
PSI_Mo × Age → NW_Mo	0.512	0.012
MSI_Fa × Age → NW_Fa	0.190	0.525

PSI: Paternal sleep involvement; MSI: Maternal sleep involvement; Fa: Reported by father; Mo: Reported by mother; NW: Number of nocturnal awakenings.
